# Hangry in the field: An experience sampling study on the impact of hunger on anger, irritability, and affect

**DOI:** 10.1371/journal.pone.0269629

**Published:** 2022-07-06

**Authors:** Viren Swami, Samantha Hochstöger, Erik Kargl, Stefan Stieger

**Affiliations:** 1 School of Psychology and Sport Science, Anglia Ruskin University, Cambridge, United Kingdom; 2 Centre for Psychological Medicine, Perdana University, Kuala Lumpur, Malaysia; 3 Department of Psychology and Psychodynamics, Karl Landsteiner University of Health Sciences, Krems an der Donau, Austria; California State University San Marcos, UNITED STATES

## Abstract

The colloquial term “hangry” refers to the notion that people become angry when hungry, but very little research has directly determined the extent to which the relationship between hunger and negative emotions is robust. Here, we examined associations between everyday experiences of hunger and negative emotions using an experience sampling method. Sixty-four participants from Central Europe completed a 21-day experience sampling phase in which they reported their hunger, anger, irritability, pleasure, and arousal at five time-points each day (total = 9,142 responses). Results indicated that greater levels of self-reported hunger were associated with greater feelings of anger and irritability, and with lower pleasure. These findings remained significant after accounting for participant sex, age, body mass index, dietary behaviours, and trait anger. In contrast, associations with arousal were not significant. These results provide evidence that everyday levels of hunger are associated with negative emotionality and supports the notion of being “hangry”.

## Introduction

During the PyeongChang Winter Olympics, American snowboarder Chloe Kim tweeted about her breakfast: “Wish I finished my breakfast sandwich but my stubborn self decided not to and now I’m getting hangry” [1]. Kim’s experience of being hangry–a portmanteau of hungry and angry–would seem to a common one: the term has entered common colloquial use, at least in the English language, and many people seem to be aware that the state of being hungry can have an effect on both emotional experiences and behaviour [2]. More specifically, both conceptual [3] and historical [4] accounts suggest that hunger often leads to negative emotions, including anger and irritability. Yet, despite this, surprisingly little research has focused on the experience, manifestation, and consequences of being hangry, particularly in everyday settings. To rectify this oversight, we report on the results of the first experience sampling study on the emotional outcomes of hunger.

### On being hangry

The state of being hungry is known to affect emotions and judgements in many different domains [5, 6], including experiences of anger and irritability. In many non-human species, for instance, food deprivation has been causally observed to increase motivations to engage in escalated and persistent aggression to gain food resources [7, 8]. Likewise, in humans, it is often assumed that hunger evokes negative emotions, such as anger, irritability, and rage, but the evidence base is somewhat equivocal. Early cross-sectional studies, for instance, linked hunger with feelings of restlessness, nervousness, and irritability [9], as well as behavioural difficulties in children [10], but operationalised emotional outcomes in different ways. More recently, some studies have investigated whether short-term fasting has an impact on mood and affect, but findings have been equivocal [11–13].

Perhaps the largest body of evidence comes from experimental studies showing that low blood glucose levels increase impulsivity, anger, and aggression [14–18]. These studies were informed by the notion of *ego depletion* [16, 19], which suggests that negative, high-arousal emotions and aggression are more likely to occur when hungry because individuals are unable to exercise self-regulation and self-control when glucose levels are low. However, scholars have questioned whether human brain functioning is substantively impaired by small reductions in blood glucose [20]. Moreover, studies with more powerful designs than the original studies assessing the theory of ego depletion have recorded mixed findings [21–23] and the theory itself has been critiqued as being overly rigid, with insufficient attention paid to the context in which glucose depletion occurs [24].

The importance of the context in which hunger occurs is underscored by a series of laboratory-based studies by MacCormack and Lindquist [3]. In two studies, these authors showed that hungrier participants experienced ambiguous pictographs as more negative, but crucially only in contexts that they interpreted as negative. In a third study, MacCormack and Lindquist [3] also demonstrated that hunger caused participants to experience negative emotions and to negatively judge a researcher, but only when they were not aware that they were conceptualising their affective state as emotions. In explanation, MacCormack and Lindquist [3] suggested that individuals are more likely to experience “greater emotionality when hunger-induced affect is conceptualized as emotions in a given context” (p. 302); that is, hungry people are more likely to experience negative emotions (e.g., anger, irritability), depending on how the context guides conceptualisations of negative emotionality.

### Being hangry outside the lab

Although research that seeks to understand the impact of hunger on negative emotions is progressing, much of this research remains limited to laboratory-based methodologies. Although laboratory-based, experimental work is crucial in terms of being able to infer causality and to better understand mechanistic pathways [3], they are also limited in terms measurement occasions (i.e., all such studies use a pre- and post-intervention method, with no longer-term follow-up or no measurement at multiple time-points). Such studies may also be limited in terms of their ecological validity; that is, laboratory-based work may not fully replicate the experience and manifestation of hunger in everyday settings [25]. In contrast, there is now greater interest in the way in which affective experiences occur in everyday settings, especially given the variability of experiences that can be tied to situation-specific needs [26].

The experience sampling method (ESM; also referred to as ecological momentary assessment or ambulatory assessment) offers an ideal research tool to more fully understand the ways in which hunger affects emotional outcomes in individuals’ everyday lives [27, 28]. In ESM studies, participants are invited to respond to prompts to complete brief surveys on multiple, semi-random occasions throughout the day over a period of time. In this way, ESM is able to generate intensive longitudinal data in a manner not possible with traditional laboratory-based research [27, 29]. Furthermore, the method is able to provide a fuller accounting of how individuals experience hunger in their everyday lives and how such experiences are associated with negative emotional outcomes (i.e., how they are hangry in their everyday lives). In other words, ESM data have the benefit of increased ecological validity [30], allowing for an examination of naturally-occurring hunger and its impact on emotional outcomes.

### The present study

In the present study, therefore, we used ESM to examine the extent to which daily experiences of hunger are associated with negative emotional outcomes. While we acknowledge that hunger is complex, involving physiological, interoceptive, and environmental inputs [31], self-reported or subjective ratings are reliable and valid indicators of the experience of hunger [32] and afford a useful (not to mention cost-effective) assessment method in the present study. As the most direct test of a link between hunger and anger (i.e., being hangry), we assessed the extent to which self-reported levels of hunger were associated with day-to-day fluctuations in anger over a 3-week period. However, because the effects of hunger are unlikely to be unique to anger [3], we also asked about experiences of irritability and, in order to obtain a more holistic view of emotionality, pleasure, and arousal as indexed using Russell’s affect grid [33]. Based on the work of MacCormack and Lindquist [3], we hypothesised that hunger would be significantly associated with greater anger and irritability, as well as greater arousal and lower pleasure as indexed by the affect grid. To ensure that our results are robust, we also conducted analyses in which we accounted for inter-individual variation in participant sex, age, body mass index (BMI), dietary behaviour, and trait anger. We expected our hypothesised relationships to remain stable even after accounting for these additional factors.

## Method

### Participants

We expected a medium-to-small effect size of *r* = .2. Based on a conservative power calculation based on the recommendation by Twisk [34], we needed a sample size of *N* = 47 (ICC = 0.3, alpha = 5%, power = 80%, one-sided, number of assessments: 5 per day over a period of 21 days = 105 single assessments per participant). Even when assuming that 20% of bings would be missed, the required sample size only increases to *N* = 48. Nevertheless, to account for possible dropout during the field phase, we aimed for a minimum of 60 participants. If we assume a two-sided hypothesis, the necessary sample size would increase to *N* = 59 (ICC = 0.3, alpha = 5%, power = 80%, two-sided, number of assessments: 5 per day over a period of 21 days = 105 single assessments per participant) and, even if 20% of bings were missed, the required sample size would only increase to *N* = 60.

In actuality, 121 participants began the study, 76 completed at least one survey per day for 21 days (39 completed all 105 requested surveys; mean number of completed surveys was *M* = 78), and 64 participants completed the study by responding to the final questionnaire–thus exceeding our requirements. Participants were on average 29.9 years old (*SD* = 12.26, range = 18–60 years) and predominantly women (81.3%; none stated *diverse* or *do not want to answer*). Most participants were from Austria (68.8%), followed by Germany (20.3%) and Switzerland (1.6%; 9.4% stated another country). In terms of relationship status, 1.6% were divorced, 35.9% in a relationship, 43.8% single and living alone, and 18.8% were married or in a partnership. Participants had invested 14.2 years on average on their education (*SD* = 3.77) and had a mean BMI of 23.8 kg/m^2^ (*SD* = 4.37, range 15.8–36.5 kg/m^2^).

### Measures

#### Daily questionnaire

Participants were asked to complete a daily survey five times a day for 21 days. Using a Visual Analogue Scale (VAS), we examined current feelings of hunger (“How hungry are you at the moment [0 = *not hungry at all*, 100 = *very hungry*]?”), irritability (“How irritable do you feel at the moment [0 = *not irritable at all*, 100 = *very irritable*]?”), and anger (“How angry are you at the moment [0 = *not angry at all*, 100 = *very angry*]?”). VASs are widely-used to assess acute hunger [35], whereas the items on irritability and annoyance were adapted based on similarly-worded items used in earlier ESM studies [36].

In addition, we assessed core affect (i.e., pleasure and arousal) using Russell’s affect grid [33, 37]. Participants were asked to state their current emotional state (“How pleasant do you find your current state [0 = *very unpleasant*, 100 = *very pleasant*]?”) and arousal level (“What is your current arousal level [0 = *sleepy*, 100 = *high arousal*]?”) again using VASs. While arousal reflects energy and mobilisation, pleasure describes a dimension of hedonic tone [38]. Both concepts have received strong empirical support [38–40] and have been used frequently in ESM research [41, 42]. Finally, we asked about the time since last meal (“When was your last meal? [___ hours ago]”).

#### Demographics

Following the installation of ESMira (see below) and registration in the study, participants were asked to respond to a request for basic demographics, i.e., sex (coded as female, male, diverse, I do not want to answer), age in years, nationality (Austria, Germany, Switzerland, other), number of years of education (including repeating classes), current relationship status, weight (in kg), and height (in cm).

#### Final survey

Following the longitudinal phase of the study (see below), participants were asked to complete a second set of demographics in order to check the validity of the data. Furthermore, we asked several questions about their eating behaviours over the previous three weeks (e.g., frequency of main meals, snacking behaviour, healthy eating, feeling hungry, sense of satiety). Finally, we assessed the constructs described below.

#### Dietary behaviour

We used a 30-item instrument [43] consisting of four subscales: restrictive eating behaviour (DB-restrictive; 10 items; sample item: “I consciously eat less so as not to gain weight”), emotionally-induced eating behaviour–clear emotions (DB-clear emotions; 7 items; sample item: “When I am irritated, I have the desire to eat”), emotionally-induced eating behaviour–unclear emotions (DB-unclear emotions; 3 items; sample item “I always want to eat something when I have nothing to do”), and externally-determined eating behaviour (DB-external; 10 items; sample item: “I eat more than usual when I see others eating”). Items were responded to on a 5-point scale from 1 (*never*) to 5 (*always*). Cronbach α was ≥ .85 for all subscale scores (for more details, see S1 Table).

#### Anger

To assess trait anger, we used the German translation [44] of the Anger subscale from the Buss and Perry Aggression Questionnaire (BPAQ) [45], which consists of 6 items (sample item: “I have trouble controlling my temper”) rated on a 5-point scale from 1 (*do not agree at all*) to 5 (*totally agree*). Cronbach α for scores on this subscale was .81 (for more details, see S1 Table).

#### Eating motivation

We used the single-item version of the Eating Motivation Survey (TEMS) [46], which consists of 15 potential eating motivations, which were responded to on 7-point scales (1 = *strongly disagree*, 7 = *strongly agree*). This item asks “Why do you eat what you eat?” followed by 15 different motivations, such as appetite, habit, hunger, for health reasons, low effort, to please me, for traditional reasons (e.g., feast), for ethical reasons (e.g., fair trade), and so forth.

#### ESMira research application

For project administration and data collection, the ESM software *ESMira* was used. ESMira offers a wide repertoire of functions for scientific data collection, such as the presentation and consent of the informed consent form, data security, data encryption, graphical feedback, anonymous chat function, and guaranteed anonymity through randomly generated codes. ESMira was available for both Android and iOS operating systems.

#### Procedure

The project was approved by the ethics committee at the Karl Landsteiner University of Health Sciences (EK Nr: 1071/2020). Participants were recruited by the second and third authors from their respective social networks. Recruitment took place via a link that led potential participants to a website presenting general information, procedures, and a request for informed consent. Individuals who elected voluntarily to participate filled in a short contact form (email, name). After receipt of the email by study staff, potential participants received an email with a link containing download instructions for ESMira, which then managed the study protocol. Furthermore, the study was not open to everyone; that is, participants were given a study-specific code in order to register for the particular study on ESMira. After downloading ESMira and opening up the app, participants had to provide digital consent in order to be successfully registered into the study. Following this, participants received a first signal (i.e., bing or notification) after a minute, which invited them to complete the first demographic survey.

Data collection took place in participants’ everyday environments (work, home, university, etc.). During the 3-week study, participants received five daily notifications on their smartphones from ESMira (i.e., in-app reminders) to complete the longitudinal part of the study. Three were sent at fixed times well before daily main meals (breakfast, lunch, dinner) in order to have time-points where hunger is likely heightened. Because we could not ask participants for their individual meal times without revealing the research hypothesis, we fixed these time-points at 8 a.m., 12 p.m., and 6 p.m. Furthermore, we used two random notifications between 9 a.m. and 11 a.m. as well as 1 p.m. and 5 p.m. Notifications were not deleted after a certain time-frame and we did not use reminders. After 21 days, the study was completed and a notification was sent asking participants to complete the final survey. Finally, a notification was sent with information about how to claim a €5 incentive.

#### Transparency and openness

Our de-identified data along with the analysis scripts and all materials are posted at https://osf.io/amz7v/. We used *R* [47] to conduct all statistical analyses using the *lme4* [48] and *sjstats* packages [49]. The study design and its analyses were not pre-registered.

#### Statistical analyses

Random-intercept, random-slope multi-level regression analyses were calculated to analyse the effects of hunger, participant sex, age, BMI, dietary behaviours, and trait anger on irritability, anger, pleasure, and arousal. Multi-level models account for the nested design of our study with measurement occasions (level 1) nested within persons (level 2). Because multi-level models can handle missing values, we did not apply any missing value imputation procedures. All level 1 predictors were centred within participants (cwc-approach) [50] and level 2 predictors were grand-mean centred (i.e., cgm) except for participant sex [51, 52]. Furthermore, following the cwc-approach of Curran and Bauer [50], the person-mean (i.e., pm) of hunger was added as a further level 2 predictor in order to separate within- and between-subject variance of hunger.

We first ran a baseline model without any predictors to calculate intraclass correlation coefficient (ICC) values (see Table 1). Next, we ran random-intercept random-slope models as depicted below. In all calculated models, a warning was presented about overly high eigenvalues. Standardising the hunger variable fixed the problem. Nevertheless, comparing models with and without standardisation revealed no difference in significance and only very minor differences in *b*-values. For ease of interpretation of the *b*-values, we report on the unstandardised calculation here.

**Table 1:** Results of the multi-level analyses.

	Fixed							Random	
	Coeff.	*B*		*CI*	*SE*	*t*		Coeff.	*SD*
**Irritability**									
Intercept (Reference)	β_00_	-4.26		-13.26 – 4.73	4.59	-0.93		*r*_0*i*_	12.06
Within-person									
Hunger.cwc	β_10_	0.16		0.12 – 0.21	0.02	6.78***		*r*_1*i*_	0.18
Between-person									
Sex (female)	β_01_	8.74		1.52 – 15.98	3.69	2.37*			
Age.cgm	β_02_	0.05		-0.20 – 0.30	0.13	0.40			
BMI.cgm	β_03_	-0.15		-0.79 – 0.50	0.33	-0.45			
DB-restrictive.cgm	β_04_	1.81		-1.65 – 5.28	1.77	1.03			
DB-clear emotions.cgm	β_05_	-3.35		-6.73 – 0.04	1.73	-1.94^+^			
DB-unclear emotions.cgm	β_06_	1.21		-2.20 – 4.61	1.74	0.70			
DB-external.cgm	β_07_	0.35		-4.00 – 4.71	2.22	0.16			
BPAQ-anger.cgm	β_08_	3.84		0.43 – 7.26	1.74	2.21*			
Hunger.pm	β_09_	0.51		0.33 – 0.70	0.10	5.42***			
*R*^2^_conditional_ = 56%, *R*^2^_marginal_ = 19%, AIC = 53966, BIC = 54068, ICC = .44									
	Fixed							Random	
	Coeff.	*B*		*CI*	*SE*	*t*		Coeff.	*SD*
**Anger**									
Intercept (Reference)	β_00_	3.92		-4.91 – 12.75	4.50	0.87		*r*_0*i*_	10.58
Within-person									
Hunger.cwc	β_10_	0.10		0.06 – 0.14	0.02	5.07***		*r*_1*i*_	0.14
Between-person									
Sex (female)	β_01_	2.21		-4.99 – 9.41	3.67	0.60			
Age.cgm	β_02_	0.10		-0.14 – 0.35	0.12	0.82			
BMI.cgm	β_03_	-0.78		-1.42 – -0.14	0.32	-2.40*			
DB-restrictive.cgm	β_04_	2.23		-1.20 – 5.66	1.75	1.28			
DB-clear emotions.cgm	β_05_	-0.62		-3.99 – 2.75	1.72	-0.36			
DB-unclear emotions.cgm	β_06_	0.24		-3.14 – 3.61	1.72	0.14			
DB-external.cgm	β_07_	1.13		-3.19 – 5.45	2.20	0.51			
BPAQ-anger.cgm	β_08_	2.64		-0.74 – 6.03	1.73	1.53			
Hunger.pm	β_09_	0.32		0.13 – 0.50	0.09	3.38**			
*R*^2^_conditional_ = 48%, *R*^2^_marginal_ = 14%, AIC = 52923, BIC = 53025, ICC = .41									
	Fixed							Random	
	Coeff.	*B*		*CI*	*SE*	*t*		Coeff.	*SD*
**Pleasure**									
Intercept (Reference)	β_00_	72.99		60.73 – 85.25	6.26	11.67***		*r*_0*i*_	13.25
Within-person									
Hunger.cwc	β_10_	-0.11		-0.15 – -0.08	0.02	-6.42***		*r*_1*i*_	0.11
Between-person									
Sex (female)	β_01_	-2.86		-12.98 – 7.26	5.16	-0.55			
Age.cgm	β_02_	-0.09		-0.43 – 0.25	0.17	-0.53			
BMI.cgm	β_03_	-0.37		-1.25 – 0.52	0.45	-0.81			
DB-restrictive.cgm	β_04_	0.29		-4.49 – 5.06	2.44	0.12			
DB-clear emotions.cgm	β_05_	0.79		-3.94 – 5.52	2.41	0.33			
DB-unclear emotions.cgm	β_06_	-0.81		-5.53 – 3.90	2.41	-0.34			
DB-external.cgm	β_07_	-1.50		-7.54 – 4.54	3.08	-0.49			
BPAQ-anger.cgm	β_08_	-2.42		-7.15 – 2.32	2.41	-1.00			
Hunger.pm	β_09_	-0.31		-0.57 – -0.05	0.13	-2.32*			
*R*^2^_conditional_ = 44%, *R*^2^_marginal_ = 6%, AIC = 59435, BIC = 59537, ICC = .37									
	Fixed							Random	
	Coeff.	*B*		*CI*	*SE*	*t*		Coeff.	*SD*
**Arousal**									
Intercept (Reference)	β_00_	45.06		35.15 – 54.97	5.06	8.91***		*r*_0*i*_	10.60
Within-person									
Hunger.cwc	β_10_	0.02		-0.02 – 0.06	0.02	0.94		*r*_1*i*_	0.14
Between-person									
Sex (female)	β_01_	-0.91		-9.07 – 7.26	4.16	-0.22			
Age.cgm	β_02_	-0.03		-0.30 – 0.25	0.14	-0.20			
BMI.cgm	β_03_	0.10		-0.61 – 0.82	0.37	0.28			
DB-restrictive.cgm	β_04_	1.71		-2.15 – 5.57	1.97	0.87			
DB-clear emotions.cgm	β_05_	-1.49		-5.30 – 2.32	1.94	-0.77			
DB-unclear emotions.cgm	β_06_	0.14		-3.66 – 3.95	1.94	0.07			
DB-external.cgm	β_07_	-3.61		-8.49 – 1.26	2.49	-1.45			
BPAQ-anger.cgm	β_08_	0.59		-3.23 – 4.42	1.95	0.30			
Hunger.pm	β_09_	0.21		0.01 – 0.42	0.11	1.99^+^			
*R*^2^_conditional_ = 29%, *R*^2^_marginal_ = 4%, AIC = 61066, BIC = 61168, ICC = .30									

Reference category for sex was male

^+^*p* < .10

*p < .05

**p < .01

***p < .001. ICC of the null model. CI = 95% Confidence Interval, DB = Dietary Behavior, BPAQ = Buss and Perry Aggression Questionnaire, cgm = grand mean centered, pm = person mean, cwc = centered within cluster (i.e., participants).

After a visual inspection of the distribution of variables, irritability and anger were found to follow a negative binomial distribution. Therefore, we additionally calculated a generalised linear mixed-effects model, first by using the *glmer*.*nb* function in *lme4* to get the dispersion parameter θ. With this, a generalised linear mixed-effects model was calculated using the *MASS* package [53] to define a negative binomial family for analyses. In both cases, significant results mainly stayed unchanged. Only the sex-specific effect for irritability did not reach statistical significance and the effect of trait anger was attenuated (only 10% significance level). Furthermore, for anger, only the intercept failed to reach statistical significance; all the other predictors did not change in significance level in the GLM model compared to the Gaussian model (see S2 Table). Again, to keep the models simple, we used the Gaussian model for all multi-level models.

Furthermore, we analysed whether a 3-level model (level 1: daily assessments, level 2: day of assessment, level 3: participants) fitted the data better than a 2-level model. For all dependent measures, the 3-level model did not present significantly better fit, except for arousal. Because the pattern of results (i.e., significance) here was stable, we only present the results of the more parsimonious 2-level model. Additionally, we analysed possible cross-level interactions between level 2 and level 1 variables. Almost all models revealed a singular fit (i.e., random variance close to zero). Of the 32 analysed cross-level interactions, only four reached statistical significance (Hunger.cwc * DB-clear emotions.cgm on irritability; Hunger.cwc * Sex and Hunger.cwc * DB-unclear emotions.cgm on Pleasure; Hunger.cwc * DB-external.cgm on activation). In order to avoid the dangers of overfitting and for the sake of a parsimonious model, we did not include cross-level interactions in the final model. This additionally raises the power of the design, because some participants did not complete either the demographic variables or the final questionnaire. The final model is displayed below:

Level 1 (within person): (Irritability, Anger, Pleasure, Activation)_ti_ = π_0i_ + π_1i_ Hunger.cwc_ti_ + *e*_ti_

Level 2 (between persons): π_0i_ = β_00_ + β_01_ Sex(female) + β_02_ Age.cgm + β_03_ BMI.cgm + β_04_ DB-restrictive.cgm + β_05_ DB-clear emotions + β_06_ DB-unclear emotions + β_07_ DB-external + β_08_ BPAQ-anger.cgm + *r*_0i_

Level 2 (between persons): π_1i_ = β_10_ + *r*_1i_

ICCs for the four dependent measures are presented in Table 1. In general, all measures showed fairly high variation *within* participants, which is reflected by relatively low ICC values, i.e., 29.8% to 44.2% of variance.

We used *R*^2^_GLMM_ [54, 55] as a measure of explained variance, which can be interpreted like the traditional *R*^2^ statistic in regression analyses. *R*^2^_marginal_ represents the proportion of variance explained by the fixed factors alone, and *R*^2^_conditional_ the proportion of variance explained by both fixed and random factors. Additionally, following Nakagawa and Schielzeth [55], we also included AIC and BIC as information criteria indices.

We also asked about the time since the last meal to have an indirect measure of hunger (i.e., the greater the time since the last meal, the higher the probability of hunger). Unfortunately, participants who skipped breakfast indicated that their last meal was the day before, which led to a bipartite distribution. Correcting the values larger than eight (the average duration of sleep) by the value 8 led to a uniform distribution. Although the correlation with hunger was substantial (*r*_sp_ = .429, *df* = 8476, *p* < .001), this was procedure was still subjective and, therefore, we excluded that variable from further analyses.

## Results

### Validity check

We compared the demographic data collected at the beginning and end of the study. In terms of participant sex, we only found one mismatch (changed from female to male). Participant age was again very accurate–in only two cases did age differed by one year. Nationality was very accurate (no mismatch), as well as relationship status (only three participants changed their status: one from being in relationship to single, one from widowed to being in a relationship, and one from being in a relationship to widowed). Furthermore, weight, height, and BMI were all highly consistent (*r* = .995, *r* = .990, and *r* = .989 respectively, all *p*s < .001). Only the number of years spent on education was less accurate, despite a strong correlation still being found (*r* = .82, *p* < .001); it may be that participants found it difficult to estimate the number of years they had invested in their education.

### Descriptive statistics and preliminary analyses

In the final questionnaire, we asked about participants’ eating behaviour during the previous three weeks. In total, 58% stated they usually had breakfast, 78% had lunch, 84% had dinner, and 48% snacked in between main meals. In addition, 9% got up at night to eat something. The majority (53%) paid attention to a healthy diet very often or always, and 55% paid attention to their hunger pangs. The main motivation to eat was hunger and because participants liked the meal. For detailed results of the eating motivation data, see S1 Fig.

Interestingly, only 23% stated that they knew when they were full and then stopped eating. In total, 63% were sure they could tell when they were full, but still continued to eat from time-to-time. A small number (4.7%) stated that they did not feel when they were full and therefore oriented their eating behaviour based on the size of the meal. Nine per cent of participants stated frequent overeating because of not feeling satiated, and 13% stated they ate when they were stressed, upset, angry, or bored. Finally, 88% said their eating behaviour in the previous three weeks was no different from their usual eating behaviour.

Because we had a substantial dropout during the data collection phase, we compared the demographics of dropouts to those who completed the final questionnaire at the end of the study. We did not find any statistical differences in terms of participant sex, χ^2^ = 3.477, *p* = .176, age, *t*(111) = 1.513, *p* = .133, relationship status, Cramer *V* = .239, *p* = .083, years of education, *t*(107) = 0.054, *p* = .957, and weight, *t*(112) = 0.729, *p* = .468. A significant difference was found only on height, *t*(114) = 2.220, *p* = .028, *d* = -0.41 (dropouts were 3.55 cm taller).

Furthermore, we analysed how strong the feeling of hunger was at our time-based sampling assessment points. As expected, in the morning (8 to 9 a.m., fixed; *M* = 31.5 *SD* = 27.7, 95% CI, 29.9, 33.0), at noon (12 a.m. to 1 p.m., fixed; *M* = 30.9 *SD* = 28.7, 95% CI, 29.4, 32.3) and in the evening (6 to 7 p.m., fixed; *M* = 32.9 *SD* = 29.1, 95% CI, 31.5, 34.4), feelings of hunger were higher than at the time frames between the main meals (9 to 11 a.m., random time-point, *M* = 27.8 *SD* = 25.2, 95% CI, 26.6, 28.9; 1 to 5 p.m., random time-point, *M* = 25.1 *SD* = 25.1, 95% CI, 24.0, 26.2). Furthermore, looking at the ratio within- vs. between-subjects (i.e., ICCs) for hunger at the five particular assessment points, we see a constant decline over the course of the day (.520, .340, .347, .222, .249). This means that, within subjects in the morning, the feelings of hunger were rather stable but become larger during the day (i.e., in the evening, the variance of hunger *within* participants is largest).

### Hypothesis-testing

As hypothesised, we found that hunger was associated with greater anger, irritability, and lower pleasure (see Table 1). Interestingly, both (ipsatised) state hunger (i.e., momentary self-reports of hunger corrected for trait hunger) and trait hunger (i.e., mean of state hunger over the previous three weeks) had predictive value in this regard. In general, associations between hunger and these dependent measures persisted even when controlling for participant sex, age, BMI, dietary behaviour, and trait anger. On the other hand, hunger was not associated with arousal (except for trait-like 3-week stable hunger at a 10% significance level), just as with all other predictors for arousal. This was interesting because one would expect hunger to lead to greater arousal. Therefore, we analysed the affect grid in more detail to see how arousal and pleasure interact with hunger–which we return to below (section Russell’s affect grid).

#### Compound measure–negative affect

Furthermore, because the results on anger, irritability, and pleasure revealed almost the same pattern with regards to significant predictors, we calculated a compound measure of these three concepts. This is also suggested by an EFA showing a one-factor solution (eigenvalue criterion) with an eigenvalue of 2.1 (70.8%). This also allowed us to calculate within- and between-subject reliabilities. We applied Generalizability Theory Analysis (GTA; [56, 57]) by using the *multilevel*.*reliability* function in the *psych* package in *R* [47] to analyze the reliability of this new measure and found satisfactory within-person reliability (*R*_C_ = .76) and excellent between-person reliability (*R*_kR_ = .98). This suggests reliable assessment of both within-person changes and inter-individual differences. The multi-level model showed a similar pattern of significant predictors: again state- and trait-hunger were the strongest predictors of the compound measure, which can be seen as an indicator of negative affect (high anger, high irritability, low pleasure; for details, see S3 Table in the online supplement).

#### Control for autoregression

Because autoregression is a common phenomenon in ESM designs, we also recalculated all models by (a) including a time variable into the model, and; (b) calculating multi-level models by accounting for autoregression effects (i.e., *corAR1* using the *lme* function in the *nlme* package in *R*). Although in two of the four multi-level models the time variable reached significance (irritability, activation), all effects were of a small effect size (all |*B*|s < 0.06). Additionally, multi-level models accounting for autoregression did not reveal any different pattern of significance compared to multi-level models without accounting for AR, except for a significant hunger.pm predictor for activation, which now reached significance, though it had already reached borderline significance (*p* < .10) in the original analysis (detailed results omitted for brevity).

#### Russell’s affect grid

**Fig 1:**
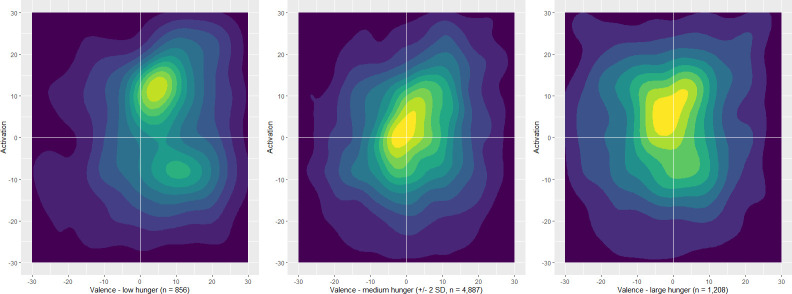
Russell’s affect grid using ipsatised data.

As can be seen in Fig 1, ratings of participants with low hunger (i.e., satiated) were focused in the upper right square (see the centre of the heat map), which means greater activation and a rather positive valence (i.e., excitement/pleasure). Ratings of participants with moderate hunger (i.e., +/- 2 *SD* around the person-specific mean ratings of the ipsatised state hunger) were focused in the middle, that is, moderate activation and average valence (i.e., pleasure). On the other hand, ratings of participants with high hunger were focused in the upper squares (i.e., activated) but have a lower valence (i.e., negative feelings, low pleasure) compared to the other two groups. Also interesting is the area of the heatmap: from low to high hunger, the centre becomes more focused (i.e., represented by a more intense yellow colour tone) and the overall area of the heat map more diverse. In short, this might indicate that the association of hunger and activation is non-linear, which could be the reason for the non-significant finding in the multi-level models. Even so, a curve estimation analyses showed that a quadratic function did not fit the data better than a linear one (both *R*^2^ similar, and the *b*-value of the quadratic term ~0; detailed results omitted for brevity).

## Discussion

In the present study, we examined associations between daily fluctuations in self-reported hunger and the experience of negative emotions. In broad outline, our results suggest that hunger is associated with stronger feelings of anger and irritability–as pointed to by the colloquial term “hanger”–as well as lower ratings of pleasure (though associations with arousal were more equivocal). This was the case when we considered each of these constructs individually, as well as when we considered a compound measure combining assessments of anger, irritability, and pleasure. Interestingly, our results suggested that both everyday variations in hunger, as well as mean hunger levels over the previous three weeks, were predictive of negative emotions. Furthermore, the effects were of a substantial size. For example, hunger explained 56% of the variance in irritability (see Table 1). This effect remained large even when only hunger centred-within-participants and person-mean hunger was included in the model (*R*^2^ = 52%). *R*^2^ values for anger and pleasure were of a similarly substantial size (48% and 44%, respectively; see Table 1). Overall, then, the present results contribute to the body of research suggesting that levels of hunger (or blood glucose levels) can affect feelings of anger and irritability [3, 14–18]. The novelty of our research, however, is in being able to show how everyday experiences of hunger (i.e., outside the laboratory) are associated with negative emotions.

Our results cannot speak to the distinction between the theory of ego depletion (i.e., that negative emotions are triggered by limited self-control as a result of low blood glucose levels) and context-dependent conceptualisations of negative emotionality. The former offers perhaps the most parsimonious explanation of our results: in this view, participants would have been expected to be less able to exercise self-regulation and self-control when hungry, thereby triggering negative emotions such as anger [16]. Our data and research design do not allow us to discount this possibility, although it should be noted that this model of self-control has been critiqued in light of large-scale replicated that have provided, at best, weak supporting evidence [22, 58]. Instead of conceptualising the hunger-negative emotions link as one arising from a lack of self-control, recent research suggests it may be more accurate to frame it in terms of the ways in which emotions are conceptualised as negative in specific situations [3].

More specifically, MacCormack and Lindquist [3] suggested that individuals experience instances of greater emotionality of multiple types (e.g., anger, irritability) when hunger-induced affect is conceptualised as emotions within specific contexts. Applied to our findings, it might be suggested that the experience of hunger is translated into negative emotions via a range of everyday situational cues and contexts that are perceived negatively. Indeed, our results showed that hunger was associated with a general feeling of lower pleasure, as indexed by Russell’s affect grid. In turn, various situational cues–such as interpersonal interactions, heat, even being prompted to complete a survey–may help individuals to make meaning of their lower pleasure by ascribing their feelings to negative emotional categories, such as anger and irritability. In other words, hunger may not automatically lead to negative emotions, but given that inferences about the meaning of affect tend to be relatively automatic and unconscious [59, 60], it may not take much for hungry individuals to experience anger and irritability.

Importantly, we found that the associations between hunger and negative emotionality were stable even after controlling for demographic factors (participant age, sex), BMI, dietary behaviour, and trait anger. This provides preliminary evidence that the link between hunger and negative emotions may be relatively robust across different social identity groups. Moreover, our results showed that negative emotions–irritability, anger, and lower pleasure–were predicted by both day-to-day fluctuations in hunger, as well as mean levels of hunger over the previous three weeks. We believe this is the first time that a link with negative emotions has been demonstrated with two different forms of self-reported hunger, suggestive that the link may be fairly robust.

In contrast, our results suggest that hunger was not significantly associated with levels of arousal. Although we suspected this may have been because the relationship between hunger and arousal was non-linear, further testing indicated that a quadratic function did not fit the data better than a linear function. Based on our results, it may be argued that it is the combination of negative states and high arousal that is linked to high levels of hunger, rather than arousal *per se*. This may also help explain why high arousal states, such as anger, in our study showed a significant relationship with self-reported hunger. More broadly, the null effect in relation to arousal is consistent with the findings of MacCormack and Lindquist [3]: to the extent that mood-congruency and attribution effects matter for determining when hunger translates to emotional outcomes, then arousal on its own may not matter as much as valence-contextualised arousal (i.e., where a negative psychological or situational context provides an impetus to conceptualise the hunger-induced arousal as an emotional state related to the situational context).

### Limitations and future directions

A strength of the present study, particularly compared to previous laboratory-based work, is our longitudinal experience sampling design. By its very nature, this methodology is able to overcome a commonplace issue with cross-sectional studies (i.e., simple between-subject view), which is that they do not always generalise to cross-temporal studies (i.e., within-subject view) and *vice versa*–a phenomenon that has been referred to as ecological fallacy or Simpson’s paradox [61, 62]. By examining the outcomes of hunger more intensively and longitudinally, as well as in everyday contexts, our study makes a unique and novel contribution to the extant literature. That is, our work provides a more ecologically valid assessment of the associations between hunger and emotional outcomes than previous studies.

Nevertheless, the results of the present study should be considered in light of a number of limiting factors. First, because we had to balance breadth of assessment with participant burden, we were unable to measure the specific situational contexts that participants found themselves in. Such data may help future researchers to disentangle more precisely whether some situations (e.g., being alone versus being with others, being at work versus at leisure) are more likely to result in anger and irritability than others. In a similar vein, we measured anger and irritability using single-item measures. Although this is a common method in ESM studies [36], this meant we were unable to capture potential nuances in these affective experiences. Also of relevance, in order to minimise participant burden, we only included measures of anger, irritability, pleasure, and arousal. Of course, there are many other emotional states that could have been measured, such as excitement, contentment, and boredom. Inclusion of a wider range of emotional states in future work help to elucidate the link between hunger and arousal (e.g., by examining associations between hunger and emotions that are neither negative nor high arousal).

Likewise, we did not obtain objective physiological measures to estimate participants’ actual hunger, such as salivary alpha-amylase, glucose, and the gut hormone ghrelin [63, 64]. This is important because subjective assessments of hunger may not be the only way that emotional and behavioural outcomes are affected; rather, it is likely that physiological and neural changes underlying feelings of hunger also induce negative emotional states [65]. Indeed, recent work has suggested that physiological hunger (as assessed using levels of alpha-amylase), but not self-reported hunger, significantly affected different types of choice behaviours [66]. Nevertheless, the absence of physiological markers of hunger in our study design may not be a major limiting factor, particularly as self-reports of hunger (i.e., how participants subjectively experience their levels of hunger) are meaningful in the context of emotionality. That is, because self-reported hunger likely depends on an awareness of hunger cues, it can perhaps be assumed that it reflects the extent to which physiological effects of hunger have translated into awareness and attributional processes. As such, self-reported hunger remains valuable in its own right, especially as hunger ratings are reliable both when made immediately [67] and after several days when tested under similar conditions [68].

Also problematic is our assumption that hunger levels increase linearly and that VAS scores can be treated as “pure” quantitative ratio scales [69]. Furthermore, we did not ask participants about possible exclusion criteria, which may have affected our results, such as Type 1 or Type 2 diabetes or a hypoglycaemia diagnosis. Individuals experiencing these conditions may have reacted differently to the questions posed because their feelings of hunger may have been experienced differently. Nevertheless, we assume that this would only have affected a small minority of participants, if at all. Finally, the absence of hunger is not the same as satiety, that is, if a participant stated on the VAS a low level of hunger (close to 0), this does not necessarily mean that this person is also satiated. Future research should assess both concepts, hunger and satiation.

In addition, our recruitment method means that our sample is unlikely to have been representative of Central European, German-speaking populations. As such, it will be important to replicate our findings with more representative and more diverse populations, so as to determine how generalisable our findings are. This is particularly important given that understandings of hunger, eating habits and styles, and attitudes toward food more generally (e.g., frequency of eating, dining rituals, the symbolic meaning of hunger) may all vary cross-culturally [70]. In future research, it would also be useful to include additional measures that may be of interest to scholars, such as eating styles and frequency of aggressive acts. Similarly, there may be value in understanding how interoceptive sensibility (i.e., the self-perceived tendency to attend to interoceptive stimuli in everyday situations) and interoceptive accuracy (i.e., the objective ability to precisely monitor one’s internal bodily state), particularly in relation to hunger and satiety [71], affect the present results.

Finally, ESM data also offer the possibility of calculating lagged effects; that is, the extent to which a variable A at time point *t* correlates with another variable B at *t*+1 when controlling for B at time-point *t*. With this design, it might be possible to determine what influences hunger at time-point *t*, and anger and irritability at time-point *t*+1, for example. Because hunger could fluctuate widely across days (range 0 to 100, *SD* = 27.2), the number of time-points per day was too low to examine lagged effects. Future research might reduce the number of days but raised the number of time-points within each day in order to analyse short-term effects of hunger on irritability, anger, pleasure, and arousal.

## Conclusion

The results of the present study suggest that the experience of being hangry is real, insofar as hunger was associated with greater anger and irritability, and lower pleasure, in our sample over a period of three weeks. These results may have important implications for understanding everyday experiences of emotions, and may also assist practitioners to more effectively ensure productive individual behaviours and interpersonal relationships (e.g., by ensuring that no one goes hungry) [2]. Although our results do not present ways to mitigate against negative hunger-induced emotions, existing research suggests that being able to label an emotion by putting feelings into words (e.g., “anger”) could help individuals to regulate those emotions. In turn, this “affect labelling” could help reduce the likelihood that hunger results in negative emotions and, by extension, behaviours. As MacCormack and Lindquist [3] have suggested, being able to label one’s affective state via emotions (e.g., “I am hangry”) could allow individuals to make sense of their experiences, but may also illuminate the best strategies to minimise those negative feelings (“I should eat”).

## Supporting information

S1 Fig Results of the Eating Motivation Survey (TEMS). (TIF)

S1 Table Reliabilities of level 2 measures. (DOCX)

S2 Table Results of the multi-level analyses assuming a negative binomial distribution for the criterion. (DOCX)

S3 Table Results of the multi-level analyses using a compound measure of irritability, anger, and pleasure for the criterion. (DOCX)
