# Supplementary figures and images for: Hangry in the field: An experience sampling study on the impact of hunger on anger, irritability, and affect
Source: PLoS One. 2022 Jul 6;17(7):e0269629. doi: 10.1371/journal.pone.0269629 (PMC9258883; doi:10.1371/journal.pone.0269629)

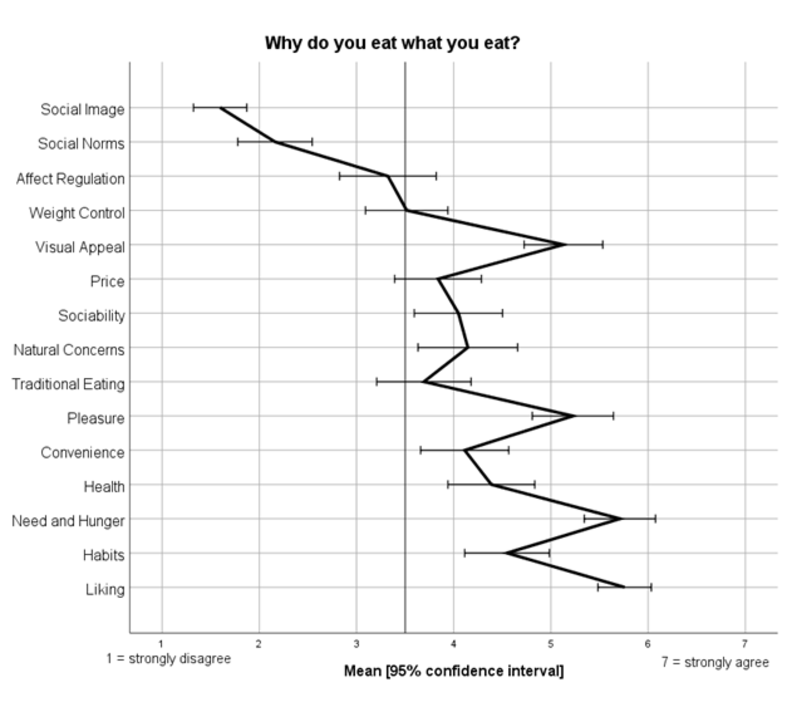

Supplement: S1 Fig — (TIF) [file pone.0269629.s001.tif]
