# Supplementary material for: Hangry in the field: An experience sampling study on the impact of hunger on anger, irritability, and affect
Source: PLoS One. 2022 Jul 6;17(7):e0269629. doi: 10.1371/journal.pone.0269629 (PMC9258883; doi:10.1371/journal.pone.0269629)
Supplement: S1 Table — (DOCX) [file pone.0269629.s002.docx]

**S1 Table. Reliabilities of Level 2 Measures**

|  | Cronbach α (95% CI) | McDonald ω_total_ (95% CI) |
| --- | --- | --- |
| DB-restrictive | .88 (.83 – .92) | .89 (.84 – .93) |
| DB-clear emotions | .90 (.86 – .94) | .91 (.87 – .94) |
| DB-unclear emotions | .85 (.83 – .94) | .88 (.83 – .93) |
| DB-external | .87 (.82 – .91) | .87 (.82 – .92) |
| BPAQ-anger | .81 (.74 – .88) | .82 (.75 – .89) |

*Note*. CI = Confidence Interval, DB = Dietary Behavior, BPAQ = Buss and Perry Aggression Questionnaire.
