# Supplementary material for: Hangry in the field: An experience sampling study on the impact of hunger on anger, irritability, and affect
Source: PLoS One. 2022 Jul 6;17(7):e0269629. doi: 10.1371/journal.pone.0269629 (PMC9258883; doi:10.1371/journal.pone.0269629)
Supplement: S2 Table — (DOCX) [file pone.0269629.s003.docx]

**S2 Table. Results of the Multi-Level Analyses assuming a Negative Binomial Distribution for the Criterion**

|  | Fixed | | | | | |  | Random | |
| --- | --- | --- | --- | --- | --- | --- | --- | --- | --- |
|  | Coeff. | *B* |  | *CI* | *SE* | *z* |  | Coeff. | *SD* |
| *Irritability* | | | | | | | | | |
| Intercept (Reference) | β_00_ | 0.99 |  | 0.20 – 1.78 | 0.40 | 2.46* |  | *r*_0_*_i_* | 0.85 |
| Within-person |  |  |  |  |  |  |  |  |  |
| Hunger.cwc | β_10_ | 0.01 |  | 0.01 – 0.01 | 0.01 | 4.97*** |  | *r*_1_*_i_* | 0.01 |
| Between-person |  |  |  |  |  |  |  |  |  |
| Sex (female) | β_01_ | 0.35 |  | -0.30 – 0.99 | 0.33 | 1.05 |  |  |  |
| Age.cgm | β_02_ | -0.01 |  | -0.03 – 0.01 | 0.01 | -0.77 |  |  |  |
| BMI.cgm | β_03_ | -0.02 |  | -0.07 – 0.03 | 0.03 | -0.70 |  |  |  |
| DB-restrictive.cgm | β_04_ | 0.25 |  | -0.04 – 0.54 | 0.15 | 1.66^+^ |  |  |  |
| DB-clear emotions.cgm | β_05_ | -0.05 |  | -0.36 – 0.26 | 0.16 | -0.33 |  |  |  |
| DB-unclear emotions.cgm | β_06_ | -0.01 |  | -0.30 – 0.30 | 0.15 | -0.02 |  |  |  |
| DB-external.cgm | β_07_ | 0.11 |  | -0.26 – 0.48 | 0.19 | 0.59 |  |  |  |
| BPAQ-anger.cgm | β_08_ | 0.25 |  | -0 04 – 0.54 | 0.15 | 1.70^+^ |  |  |  |
| Hunger.pm | β_09_ | 0.04 |  | 0.02 – 0.06 | 0.01 | 4.78*** |  |  |  |
| *R*^2^_conditional_ = 62%, *R*^2^_marginal_ = 23% | | | | | | | | | |
| *Anger* | | | | | | | | | |
| Intercept (Reference) | β_00_ | 1.46 |  | 0.71 – 2.21 | 0.38 | 3.82*** |  | *r*_0_*_i_* | 0.81 |
| Within-person |  |  |  |  |  |  |  |  |  |
| Hunger.cwc | β_10_ | 0.01 |  | <0.01 – 0.01 | 0.01 | 3.63*** |  | *r*_1_*_i_* | 0.01 |
| Between-person |  |  |  |  |  |  |  |  |  |
| Sex (female) | β_01_ | -0.11 |  | -0.73 – 0.50 | 0.31 | -0.36 |  |  |  |
| Age.cgm | β_02_ | -0.01 |  | -0.03 – 0.02 | 0.01 | -0.45 |  |  |  |
| BMI.cgm | β_03_ | -0.06 |  | -0.12 – -0.01 | 0.03 | -2.24* |  |  |  |
| DB-restrictive.cgm | β_04_ | 0.26 |  | -0.03 – 0.54 | 0.15 | 1.74^+^ |  |  |  |
| DB-clear emotions.cgm | β_05_ | 0.14 |  | -0.16 – 0.43 | 0.15 | 0.92 |  |  |  |
| DB-unclear emotions.cgm | β_06_ | -0.12 |  | -0.41 – 0.16 | 0.15 | -0.85 |  |  |  |
| DB-external.cgm | β_07_ | 0.22 |  | -0.15 – 0.58 | 0.19 | 1.15 |  |  |  |
| BPAQ-anger.cgm | β_08_ | 0.24 |  | -0.05 – 0.53 | 0.15 | 1.63 |  |  |  |
| Hunger.pm | β_09_ | 0.03 |  | 0.02 – 0.05 | 0.01 | 3.91*** |  |  |  |
| *R*^2^_conditional_ = 59%, *R*^2^_marginal_ = 22% | | | | | | | | | |

*Note*. Reference category sex was male. ^+^ *p* < .10, **p* < .05, ***p* < .01, ****p* < .001.

cgm = grand mean centered, pm = person mean, cwc = centered within cluster (i.e., participants).
