# Supplementary material for: Hangry in the field: An experience sampling study on the impact of hunger on anger, irritability, and affect
Source: PLoS One. 2022 Jul 6;17(7):e0269629. doi: 10.1371/journal.pone.0269629 (PMC9258883; doi:10.1371/journal.pone.0269629)
Supplement: S3 Table — (DOCX) [file pone.0269629.s004.docx]

**S3 Table. Results of the Multi-Level Analyses using a Compound Measure of Irritability, Anger, and Pleasure for the Criterion**

|  | Fixed | | | | | |  | Random | |
| --- | --- | --- | --- | --- | --- | --- | --- | --- | --- |
|  | Coeff. | *B* |  | *CI* | *SE* | *t* |  | Coeff. | *SD* |
| Negative Affect: compound measure (Irritability, Anger, Pleasantness[reversed]) | | | | | | | | | |
| Intercept (Reference) | β_00_ | 8.80 |  | 0.32 – 17.29 | 4.33 | 2.04* |  | *r*_0_*_i_* | 10.0 |
| Within-person |  |  |  |  |  |  |  |  |  |
| Hunger.cwc | β_10_ | 0.12 |  | 0.09 – 0.16 | 0.02 | 6.63*** |  | *r*_1_*_i_* | 0.14 |
| Between-person |  |  |  |  |  |  |  |  |  |
| Sex (female) | β_01_ | 5.75 |  | -1.19 – 12.68 | 3.54 | 1.62 |  |  |  |
| Age.cgm | β_02_ | 0.05 |  | -0.19 – 0.28 | 0.12 | 0.41 |  |  |  |
| BMI.cgm | β_03_ | -0.23 |  | -0.84 – 0.38 | 0.31 | -0.74 |  |  |  |
| DB-restrictive.cgm | β_04_ | 1.20 |  | -2.09 – 4.49 | 1.68 | 0.71 |  |  |  |
| DB-clear emotions.cgm | β_05_ | -1.48 |  | -4.72 – 1.76 | 1.65 | -0.90 |  |  |  |
| DB-unclear emotions.cgm | β_06_ | 0.39 |  | -2.85 – 3.63 | 1.65 | 0.24 |  |  |  |
| DB-external.cgm | β_07_ | 1.45 |  | -2.70 – 5.60 | 2.12 | 0.69 |  |  |  |
| BPAQ-anger.cgm | β_08_ | 2.55 |  | -0.70 – 5.81 | 1.66 | 1.54 |  |  |  |
| Hunger.pm | β_09_ | 0.37 |  | 0.19 – 0.55 | 0.09 | 4.05*** |  |  |  |
| *R*^2^_conditional_ = 51%, *R*^2^_marginal_ = 15%, AIC = 55476, BIC = 55579, ICC = .41 | | | | | | | | | |

*Note*. Reference category sex was male. ^+^ *p* < .10, **p* < .05, ***p* < .01, ****p* < .001.

cgm = grand mean centered, pm = person mean, cwc = centered within cluster (i.e., participants).
